# Anti-Inflammatory Properties of Brazilian Green Propolis Encapsulated in a γ-Cyclodextrin Complex in Mice Fed a Western-Type Diet

**DOI:** 10.3390/ijms18061141

**Published:** 2017-05-26

**Authors:** Gerald Rimbach, Alexandra Fischer, Anke Schloesser, Gerold Jerz, Naoko Ikuta, Yoshiyuki Ishida, Ryota Matsuzawa, Seiichi Matsugo, Patricia Huebbe, Keiji Terao

**Affiliations:** 1 Institute of Human Nutrition and Food Science, Food Science, University of Kiel, Hermann-Rodewald-Strasse 6, 24118 Kiel, Germany; fischer@foodsci.uni-kiel.de (A.F.); schloesser@foodsci.uni-kiel.de (A.S.); huebbe@foodsci.uni-kiel.de (P.H.); 2 Institute of Food Chemistry, Technical University Braunschweig, Schleinitzstrasse 20, 38106 Braunschweig, Germany; g.jerz@tu-braunschweig.de; 3 Graduate School of Medicine, Kobe University, 7-5-1, Kusunoki-cho, Chuo-ku, 650-0017 Kobe, Japan; naoko.ikuta@people.kobe-u.ac.jp; 4 CycloChem Bio Co., Ltd., 7-4-5 Minatojima-minamimachi, Chuo-ku, 650-0047 Kobe, Japan; yoshiyuki.ishida@cyclochem.com (Y.I.); keiji.terao@cyclochem.com (K.T.); 5 School of Natural System, Kanazawa University, Kakuma-machi, 920-1192 Kanazawa, Japan; ryoooooota6623@gmail.com (R.M.); s-matsugoh@se.kanazawa-u.ac.jp (S.M.)

**Keywords:** Brazilian green propolis, artepillin C, mice, inflammation, antioxidant defence, diet

## Abstract

Ageing is often accompanied by chronic inflammation. A fat- and sugar-rich Western-type diet (WTD) may accelerate the ageing phenotype. Cell culture studies have indicated that artepillin C-containing Brazilian green propolis exhibits anti-inflammatory properties. However, little is known regarding its anti-inflammatory potential in mouse liver in vivo. In this study, female C57BL/6NRj wild-type mice were fed a WTD, a WTD supplemented with Brazilian green propolis supercritical extract (GPSE) encapsulated in γ-cyclodextrin (γCD) or a WTD plus γCD for 10 weeks. GPSE-γCD did not affect the food intake, body weight or body composition of the mice. However, mRNA levels of the tumour necrosis factor α were significantly downregulated (*p* < 0.05) in these mice compared to those in the WTD-fed controls. Furthermore, the gene expression levels of other pro-inflammatory markers, including serum amyloid P, were significantly (*p* < 0.001) decreased following GPSE-γCD treatment. GPSE-γCD significantly induced hepatic ferritin gene expression (*p* < 0.01), which may contribute to its anti-inflammatory properties. Conversely, GPSE-γCD did not affect the biomarkers of endogenous antioxidant defence, including catalase, glutathione peroxidase-4, paraoxonase-1, glutamate cysteine ligase and nuclear factor erythroid 2-related factor-2 (Nrf2). Overall, the present data suggest that dietary GPSE-γCD exhibits anti-inflammatory, but not antioxidant activity in mouse liver in vivo. Thus, GPSE-γCD has the potential to serve as a natural hepatoprotective bioactive compound for dietary-mediated strategies against chronic inflammation.

## 1. Introduction

**Figure 1:**
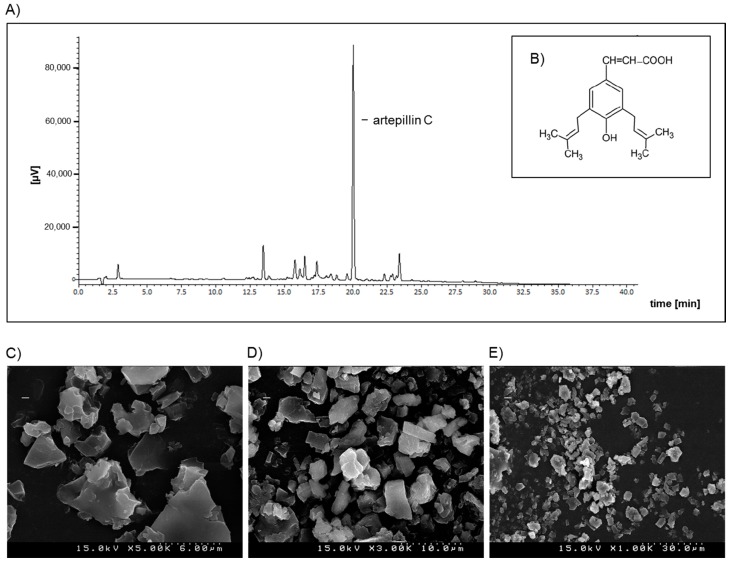
Representative HPLC chromatogram of (**A**) free artepillin C at 320 nm from the complex of green propolis supercritical extract encapsulated in γ-cyclodextrin (GPSE-γCD) and (**B**) its chemical structure; and (**C**–**E**) Morphological characterization of the GPSE-γCD complex by scanning electron microscopy. The GPSE-γCD complex was analysed at 15 kV in three different fields within each sample, and three images of each field were measured at the magnifications 1000, 3000 and 5000.

Propolis (also known as “bee glue”), which is gathered from plants and produced by honeybees to seal and protect the hive, is a chemically complex mixture of numerous bioactive compounds. Thus far, a couple of hundred molecules have been identified in propolis, and its chemical composition largely depends on its geographical origin [1,2,3,4]. Brazilian green propolis is rich in artepillin C (3,5-diprenyl-4 hydroxycinnamic acid; for its chemical structure, see Figure 1B), whereas propolis from New Zealand contains significant amounts of caffeic ester phenethyl ester (CAPE) [5,6]. Besides artepillin C, Brazilian green propolis contains also other bioactives including p-coumaric acid, baccharin, drupanin and cinnamic acid [7].

Brazilian green propolis is used as a health supplement because it exhibits the potential to stimulate immune function and wound healing and has antimicrobial and antioxidant properties [8,9]. Moreover, propolis was shown to be effective in reducing the secretion of lipopolysaccharide (LPS)-induced pro-inflammatory chemokines and tumour necrosis factor α (TNF-α) in cultured cells [10,11]. Accordingly, propolis injection has been shown to inhibit the systemic inflammatory response in acute septic shock in LPS-challenged mice [12]. However a pilot, randomized, placebo-controlled study on the effect of artepillin C-rich extract of Brazilian propolis in frequent colorectal adenoma polyp patients did not provide evidence that Brazilian propolis was effective in preventing changes occurring during early stages of colon cancer [13].

Diet and age are the major determinants of chronic inflammation. It has been consistently shown that a so-called Western-type diet (WTD) rich in fat and sugars accelerates inflammatory processes in various tissues and organs, including the liver [14,15]. Furthermore, an increasing amount of experimental evidence suggests that ageing is accompanied by dysregulated immune and chronic inflammatory responses [16,17]. Thus, dietary strategies are needed to counteract diet- and age-related chronic inflammation.

Cyclodextrins are produced by the enzymatic conversion of starch and are widely used to improve the solubility and stability of pharmaceuticals and nutraceuticals [18,19]. It has previously been shown that the encapsulation of tocotrienols [20], lipoic acid [21,22], and coenzyme Q_10_ [23] with γ-cyclodextrin (γCD, which is composed of eight monomers arranged in a cyclic ring) results in improved bioactivity in mice and humans. Furthermore, the encapsulation of propolis-derived CAPE with γCD significantly enhanced its anti-cancer properties in cultured cells and in mice [6].

Therefore, the aim of the current study was to investigate the potential anti-inflammatory and antioxidant properties of a dietary green propolis supercritical extract (GPSE) encapsulated in γCD in laboratory mice. To further promote the development of chronic inflammation in the liver, mice were fed a high-sugar, high-fat WTD in the absence and presence of GPSE-γCD.

## 2. Results

### 2.1. Body Weight, Food Intake, Blood Glucose and Body Composition

Mice were healthy throughout the 10-week feeding trial, and the dietary intervention with a WTD supplemented with either γCD or GPSE-γCD was well tolerated. Food intake and body weight over the experimental period of 10 weeks were not significantly different among the groups (Table S1).

**Figure 2:**
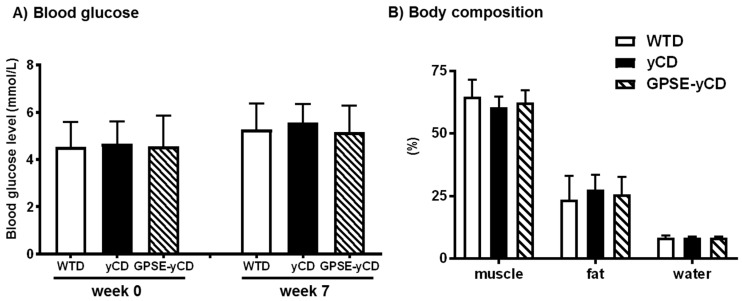
(**A**) Blood glucose and (**B**) body composition after 7 weeks in mice fed a Western-type diet (WTD), a WTD supplemented with γ-cyclodextrin (γCD) or a WTD supplemented with Brazilian green propolis supercritical extract encapsulated in γCD (GPSE-γCD). The fasting blood glucose levels in blood were measured using a glucometer at 0 and 7 weeks. Body composition was measured using a time-domain nuclear magnetic resonance technique in the live animals without using anaesthesia. The data are presented as the mean ± SD (*n* = 10 mice/diet). Statistical analyses were performed using one-way ANOVA, followed by Bonferroni correction or, in the case of inhomogeneity of variance, by Dunnett’s multiple comparison post hoc test.

Blood for glucose determination was taken at week 0 and week 7. No significant differences in the plasma glucose concentrations were observed at any time among the groups, as summarized in Figure 2A. The percentages of muscle, fat and water mass were determined in live mice at week 7. The body composition was similar among the three experimental groups (Figure 2B).

### 2.2. Inflammatory Gene Expression

**Figure 3:**
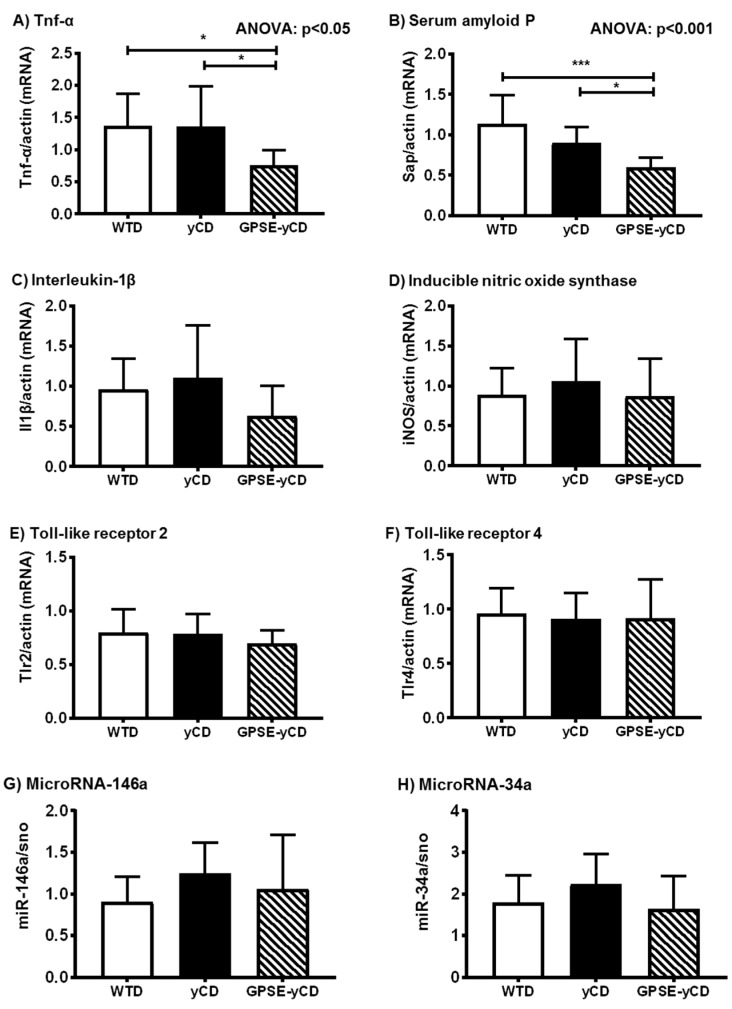
Expression levels of inflammatory genes as determined by quantitative reverse transcriptase PCR in the livers of mice fed a Western-type diet (WTD), a WTD supplemented with γ-cyclodextrin (γCD) or a WTD supplemented with Brazilian green propolis supercritical extract encapsulated in γCD (GPSE-γCD). The data are presented as the mean ± SD (*n* = 10 mice/diet). Significant differences were calculated using one-way ANOVA, followed by Bonferroni correction or, in the case of inhomogeneity of variance, by Dunnett’s multiple comparison post hoc test. * *p* < 0.05; *** *p* < 0.001. Expression levels of (**A**) TNF-α; (**B**) serum amyloid P; (**C**) interleukin-1β; (**D**) inducible nitric oxide synthase; (**E**) toll-like receptor 2; (**F**) toll-like receptor 4; (**G**) miR-146a; (**H**) miR-34a/sno are given.

Mice receiving the diet supplemented with GPSE-γCD exhibited significantly lower mRNA levels of TNF-α in the liver compared to control mice receiving the WTD only (Figure 3A). Similar to TNF-α, interleukin-1β mRNA levels were also lower in the GPSE-γCD-fed mice versus the WTD-fed controls, but the differences did not reach statistical significance (Figure 3C). Furthermore, the gene expression level of the pro-inflammatory biomarker serum amyloid P (Sap) was significantly reduced following the GPSE-γCD dietary treatment (Figure 3B). There were no differences in the mRNA levels of hepatic inducible nitric oxide synthase among the groups (Figure 3D).

The expression of inflammatory genes is partly regulated by toll-like receptors (Tlrs) and nuclear factor κ-light-chain-enhancer of activated B-cells (NF-κB) signalling. Therefore, we determined the steady-state mRNA levels of Tlr2 and Tlr4. However, under the conditions investigated, Tlr2 and Tlr4 mRNA levels remained unchanged in response to the different dietary treatments (Figure 3E,F). Additionally, levels of microRNA (miR)-146a and miR-34a, also important regulators of the inflammatory response, remained largely unaffected by the GPSE-γCD dietary treatment (Figure 3G,H).

### 2.3. Antioxidant Defence

The expression of genes encoding proteins involved in antioxidant defence and stress response pathways were determined by quantitative reverse transcriptase PCR in the livers of mice (*n* = 10 mice/diet). GPSE-γCD did not significantly affect the biomarkers of the endogenous antioxidant defence system, including glutamate cysteine ligase modifier subunit (8.53% increase compared to WTD) and catalytic subunit (19.2% decrease compared to WTD), paraoxonase 1 (17.9% decrease compared to WTD), catalase (19.7% increase compared to WTD), and glutathione peroxidase 4 (18.8% increase compared to WTD). Similarly, the mRNA level of nuclear factor erythroid 2-related factor-2 (Nrf2), which partly controls antioxidant gene expression, was not affected by GPSE-γCD (13.3% increase compared to WTD).

### 2.4. Inflammatory Protein Expression

**Figure 4:**
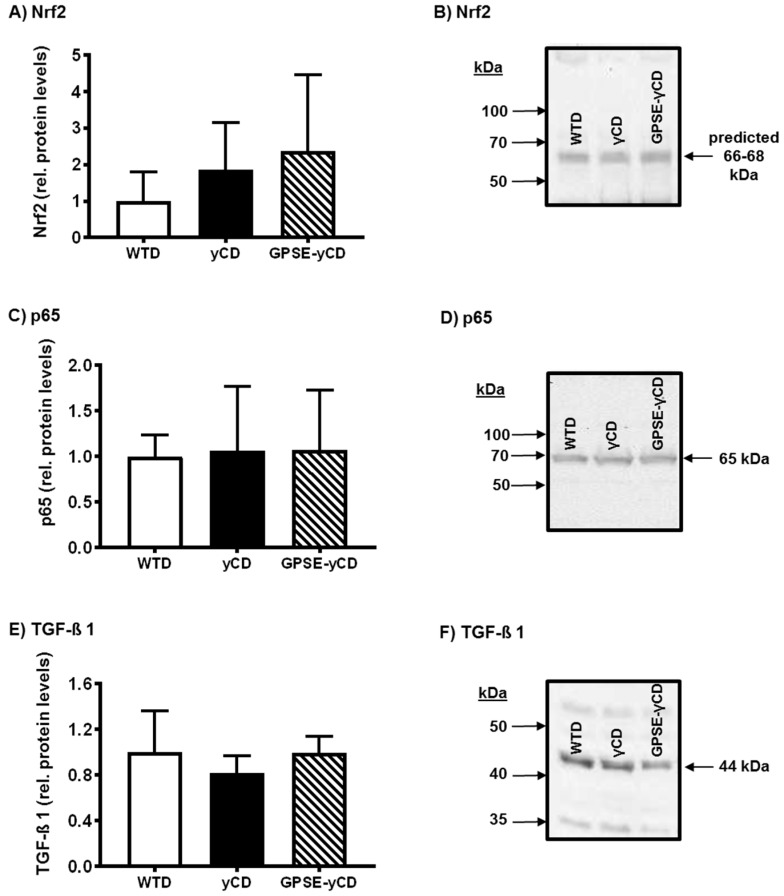
Relative expression levels of proteins as determined by Western blotting in the livers of mice fed a Western-type diet (WTD), a WTD supplemented with γ-cyclodextrin (γCD) or a WTD supplemented with Brazilian green propolis supercritical extract encapsulated in γCD (GPSE-γCD). (**A**,**C**,**E**) The relative intensities of bands were quantified by densitometry and the total protein in each lane was used as the loading control. The mean band intensity in the WTD group was set to be 1. Relative protein levels are presented as the mean ± SD (*n* = 10 mice/diet); and (**B**,**D**,**F**) Representative Western blots of nuclear factor erythroid 2-related factor-2 (Nrf2), p65 and transforming growth factor (TGF-β1) proteins detected by specific antibodies in the livers of mice, fed the different experimental diets.

Additionally to gene expression data, the expression of different proteins involved in stress response and inflammation, including Nrf2, the NF-κB subunit p65 and transforming growth factor β1 (TGF-β1), were determined. However, the relative expression of proteins or the translocation of p65 to the nucleus respectively were not significantly modulated by GPSE-γCD feeding in the livers of mice (Figure 4).

### 2.5. Ferritin, Hepcidin, and Metallothionein

**Figure 5:**
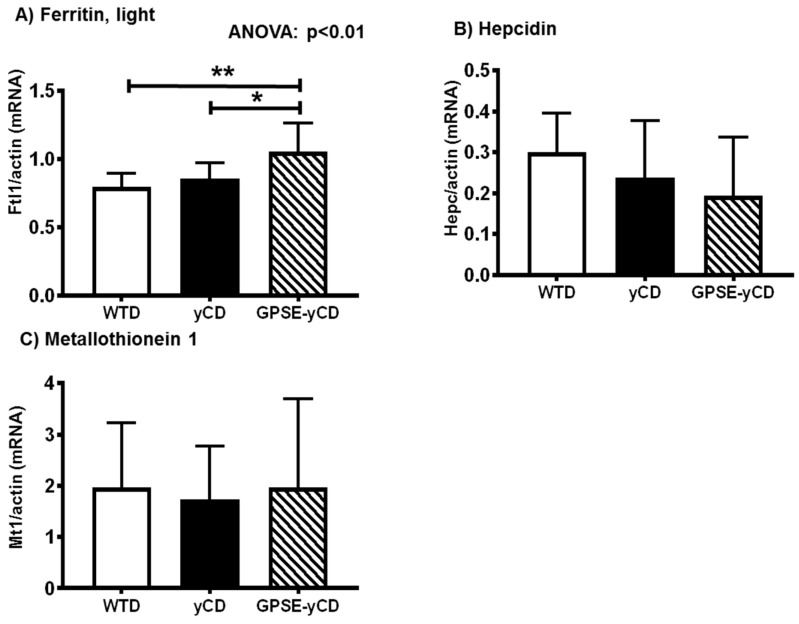
(**A**) Expression of ferritin, (**B**) hepcidin and (**C**) metallothionein 1 genes, as determined by quantitative reverse transcriptase PCR, in the livers of mice fed a Western-type diet (WTD), a WTD supplemented with γ-cyclodextrin (γCD) or a WTD supplemented with Brazilian green propolis supercritical extract encapsulated in γCD (GPSE-γCD). The data are presented as the mean ± SD (*n* = 10 mice/diet). Significant differences were calculated using one-way ANOVA, followed by Bonferroni correction, or in the case of inhomogeneity of variance, by Dunnett’s multiple comparison post hoc test. * *p* < 0.05; ** *p* < 0.01.

Interestingly, mRNA levels of ferritin were significantly higher in the GPSE-treated mice than in the controls (Figure 5A). Hepatic hepcidin and metallothionein gene expression levels were similar among the groups (Figure 5B,C).

## 3. Discussion

The liver plays a crucial role in metabolizing nutritional substrates and is centrally involved in regulating the inflammatory response to nutrients and injury [24]. Hepatic inflammation may be a characteristic of chronic high-energy intake [25]. Furthermore, our previous data suggested that mice exhibiting an accelerated ageing phenotype and fed a Western-type diet had elevated levels of hepatic biomarkers of inflammation, including Sap [26]. In the present study, GPSE-γCD downregulated both hepatic TNF-α and Sap mRNA levels in mice fed an energy-dense WTD, indicating that GPSE-γCD exerts anti-inflammatory effects. However, the underlying cellular and molecular mechanisms by which GPSE-γCD may have prevented and/or counteracted the diet-induced inflammation in this study are not completely understood.

Western-type diets have been reported to induce liver steatosis, inflammation and early dysregulation of iron metabolism in laboratory rodents. Meli and co-workers reported significantly decreased hepatic ferritin concentrations in rats fed a WTD for 8 weeks [27]. It has been suggested that ferritin acts as a cytoprotective molecule by decreasing the reactivity of redox-reactive free iron, thereby preventing chronic inflammatory conditions. In the present study, we observed an increase in hepatic ferritin mRNA levels in the GPSE-γCD-fed mice compared to the controls. Thus, it is possible that GPSE-γCD may partly exhibit anti-inflammatory activity by increasing the levels of hepatic ferritin, which in turn alters iron metabolism.

Toll-like receptors [28], microRNAs [29,30] and the antioxidant status [31] are important regulators of inflammatory processes. We analysed microRNA-146a, which has been shown to regulate inflammation through the Tlr4-mediated pathway [32] and microRNA-34a, which is also involved in the regulation of inflammatory response [33] and autophagy [34]. Furthermore the redox-sensitive transcription factor nuclear factor erythroid 2-related factor-2 (Nrf2), which confers protection against a wide spectrum of stressors, including oxidative and chemical stress [35] and partly regulates the expression of genes encoding antioxidant enzymes [36], was also evaluated. However, we did not observe differences in the levels of Tlr2, Tlr4, miR-146a, miR-34a or the expression of Nrf2 or genes encoding antioxidant enzymes (catalase, glutathione peroxidase 4, paraoxonase-1 and glutamate cysteine ligase) in GPSE-γCD-fed mice. Moreover, we analysed by Western blot analysis the expression of the Nrf2 protein, the nuclear p65 protein (a subunit of the transcription factor NF-κB, a critical regulator of many cellular processes including cell survival and inflammation [37]) and the transforming growth factor (TGF-β1) protein (which plays an important role in the pathogenesis of redox-regulated fibrosis [38]). However, feeding of GPSE-γCD to mice did not change the expression of the examined proteins. Thus, the present data suggest that the differences in inflammatory gene expression in response to GPSE-γCD are not mediated by changes in Tlr2, Tlr4, Nrf2, TGF-β1, NF-κB signalling, miR-146a, miR-34a, or endogenous enzymatic antioxidant defence mechanisms.

According to our analyses and data from the literature [4,7], artepillin C is an important constituent of Brazilian green propolis. Artepillin has been previously shown to mediate its bioactivity through the activation of p38 MAPK (mitogen-activated protein kinase) via the extracellular signal-regulated kinase (ERK) signalling pathway [39], which can partly explain its anti-inflammatory properties. Artepillin C is a relatively lipophilic molecule with a rather poor bioavailability and high susceptibility to hepatic elimination [40], therefore, we encapsulated it with γCD to improve its bioactivity. Importantly, γCD supplementation per se did not affect the inflammatory gene expression, suggesting that the anti-inflammatory properties of GPSE-γCD may be mainly attributed to the bioactive molecules present in GPSE. However, we did not measure the concentrations of artepillin C in the plasma or tissues in the present study. Future studies are needed to determine whether and to what extent the γCD encapsulation may affect the bioavailability of artepillin C and other GPSE-derived bioactive molecules. In a previous study, we encapsulated purified lipoic acid into γCD to improve its stability [21]. Unlike the lipoic acid-γCD complex, we observed a relatively variable and non-uniform particle shape and size for the GPSE-γCD complex. This finding may be related to the fact that Brazilian green propolis contains numerous different molecules [4] that participate in individual and diverse complex formation. In fact, beside artepillin C, we also identified other hydrocarbon based compounds, including phytosterols, tripenoids and beewaxes, which is in agreement with the literature [41].

## 4. Materials and Methods

### 4.1. Experimental Animals and Diets

Eight-week-old female C57BL/6NRj mice were acquired from Janvier Labs (Le Genest-Saint-Isle, France). Tap water and the experimental diets were freely available to the mice throughout the experiment. Mice were housed in groups in Makrolon cages and provided with bedding, including wood–wool, in a conditioned room (temperature, 22 ± 2 °C; relative humidity, 50–60%; 12 h light/dark cycle).

After two weeks of adaptation, the mice were split into three groups of 10 mice each (mean body weight: 19.2 ± 0.18 g). The first group of mice was fed a purified semisynthetic, energy-dense high-fat and high-sugar Western-type diet (WTD, Ssniff E15721-34) for 10 weeks (Table 1). The second group of mice was fed the WTD plus 2.2 g/kg γ-cyclodextrin (WTD+γCD, Cavamax W8, Wacker, Stuttgart, Germany), and the third group of mice was fed the WTD plus 2.3 g/kg green propolis supercritical extract encapsulated in γCD (WTD+GPSE-γCD, Fujimi Youhouen, Saitama, Japan), providing 200 mg artepillin C/kg diet. The animal experiment was conducted in accordance with the guidelines for the care and use of animals for experimental procedures with approval of the Ministry of Agriculture, Environment and Rural Areas of the State of Schleswig-Holstein (Germany) (V 242-16213/2016).

The food intake was recorded every day, and the body weight of the mice was recorded every week. After 10 weeks, the mice were euthanized by cervical dislocation, and blood was collected immediately from the heart using a heparin-coated syringe and placed on ice for 30 min. Plasma was obtained by centrifugation (3000× *g*, 10 min, 4 °C) and stored at −80 °C until analysis. The livers were removed, snap frozen in liquid nitrogen and stored at −80 °C until analysis. The liver samples for RNA isolation were removed and stored in RNAlater (Qiagen, Hilden, Germany) at −20 °C.

### 4.2. Preparation of GPSE/γ-Cyclodextrin Complex and Artepillin C for Analysis by HPLC

Green propolis supercritical extract (GPSE) was obtained from Fujimi Youhouen, Saitama, Japan. Eighty-three grams of GPSE and 250 g of γCD (dry weight: 227 g) were added to 666 mL water, and the solution was homogenized at 65 °C to obtain a colloidal suspension. The prepared suspension was diluted with water and then spray-dried.

The concentration of artepillin C in the GPSE-γCD complex was analysed by HPLC using a SunFire C18 column (5 μm, 4.6 mm I.D. × 150 mm). The mobile phase A consisted of 0.5% acetic acid, and the mobile phase B contained acetonitrile while using a gradient profile at a flow rate of 1.0 mL/min and at a temperature of 40 °C. Artepillin C (Wako Pure Chemical Ind., Ltd., Osaka, Japan) was used as an external standard. The artepillin C stock solution (0.02 mg/mL) was prepared in ethanol and filtered (Advantec Dismic-25, 0.2 μm). The GPSE-γCD complex was dispersed in ethanol, and the same volume of acetonitrile and 0.5% acetic acid were added. In the solution, artepillin C was dissociated, and the free artepillin C could be detected at 320 nm (Figure 1A). The retention time of artepillin C was approximately 20 min and the concentration of artepillin C was calculated via an external standard curve using the peak area. The stability of artepillin C in the GPSE-γCD complex was evaluated by HPLC and the recovery was found to be 97% after storage for 4 weeks at 40 °C (data not shown).

### 4.3. L C-APCI-IT-MS/MS Analysis of the Green Propolis Supercritical Extract (GPSE)

Liquid chromatography (LC) atmospheric pressure chemical ionization (APCI) ion-trap (IT) with mass spectrometry (MS/MS) fragmentation experiments of green propolis supercritical extract (GPSE) were performed in negative and positive ionization mode on an HCT-Ultra-ETD II ion-trap mass-spectrometer (Bruker Daltonics, Bremen, Germany). The chromatographic separation was achieved using a 250 × 2.0 mm column, Prontosil C18Aq (Knauer Geraetebau, Berlin, Germany) with a pre-column cartridge of the identical material. The following gradient was used with a flow rate of 0.5 mL/min (solvent A: *tert*-butylmethylether/methanol/water 4:92:4, solvent B: *tert*-butylmethylether/methanol/water 90:6:4): 0–10 min (100% A), 10–20 min (100–50% A), 20–33 min (50–0% A), 33−37 min (0–100% A), 37−45 min (re-equilibration 100% A). Injection volume was 10 μL, and the temperature of the column oven was set to 20 °C. Following APCI-IT-MS/MS, general parameters were used: drying gas nitrogen, 45 psi and 7.0 L/min; vaporizer temperature, 400 °C; fragmentation amplitude, 1 V; target mass range, *m*/*z* 500, compound stability, 80%; trap drive level, 120%; ion charge control (ICC) on; maximal ion accumulator time, 200 ms.

**Figure 6:**
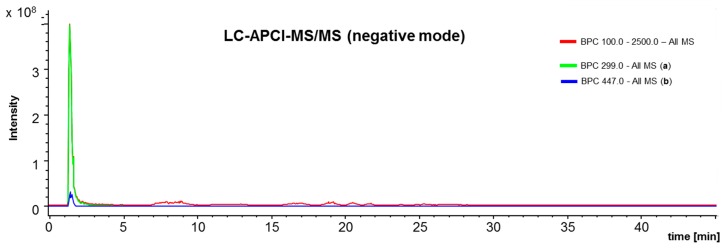
Representative liquid chromatography-atmospheric pressure chemical ionization ion-trap mass spectrometry (LC-APCI-IT-MS/MS) (negative mode) chromatogram of green propolis supercritical extract (GPSE). Scanning range was from *m*/*z* 100 to *m*/*z* 2500; ionization voltage, +900 V; end plate offset, −500 V, Trap Drive, 79.2; Octopole RF Amplitudein Volt; 187.1 Vpp; lens 2, 60 V; Cap Exit −115 V; Smart ion charge control (ICC) target, 70,000. (**a**): artepillin C, retention time (Rt) 1.5 min: [M-H]^−^ *m*/*z* 299, MS/MS: 255, 200. (**b**): (*E*)-3-(4-hydroxy-3-[(*E*)-4-(2,3-dihydro-cinnamoyl-oxy)-3-methyl-2-butenyl]-5-prenyl-phenyl)-2-propenoic acid, Rt 1.5 min: [M-H]^−^ *m*/*z* 447, MS/MS: 297, 253, 198, 149 [42].

**Figure 7:**
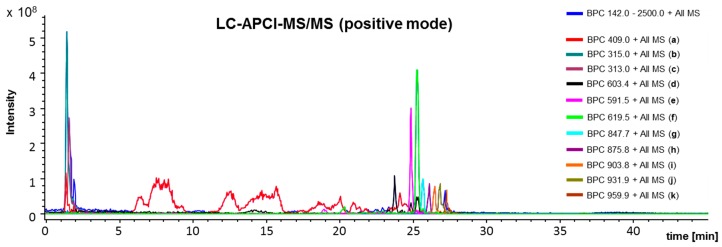
Representative LC-APCI-IT-MS/MS (positive mode) chromatogram of green propolis supercritical extract (GPSE). Scanning range was from *m*/*z* 142 to *m*/*z* 2500. ionization voltage, −3500 V; end plate offset, −500 V, Trap Drive , 63.2; Octopole RF Ampl.; 187.1 Vpp; lens 2, −60 V; Cap Exit 115 V, Smart ICCC target, 100,000. (**a**): chromatographically not sufficiently resolved components; (**b**): unknown, retention time (Rt) 1.5 min: [M+H]^+^ *m*/*z* 315.1, MS/MS 283, 273, 259; (**c**): unknown, Rt 1.7 min: [M+H]^+^ *m*/*z* 313.0, MS/MS 282, 271; (**d**): unknown, Rt 23.7 min: [M+H]^+^ *m*/*z* 603.4, MS/MS 586, 529, 364, 339, 265; (**e**): unknown, Rt 24.8 min: [M+H]^+^ *m*/*z* 591.5, MS/MS 309, 291, 254; (**f**): unknown, Rt 25.2 min: [M+H]^+^ *m*/*z* 619.1, no MS/MS fragments; (**g**): diester of palmitic acid (16:0/22-diol/16:0), Rt 25.6 min: [M+H]^+^ *m*/*z* 847.7, MS/MS 591, 291, 255 [44]; (**h**): hydroxylated palmitic acid diester, Rt 26.1 min: [M+H]^+^ *m*/*z* 875.8, MS/MS 619, 255 [44]; (**i**): hydroxylated palmitic acid diester, Rt 26.4 min: [M+H]^+^ *m*/*z* 903.8, MS/MS 647, 619, 255 [44]; (**j**): wax diester, Rt 26.8 min: [M+H]^+^ *m*/*z* 931.9, MS/MS 675.6 [44]; (**k**): wax diester, Rt 27.2 min: [M+H]^+^ *m*/*z* 959.9, MS/MS 703.7 [44]; and wax diester, Rt 27.1 min: [M+H]^+^ *m*/*z* 985.9, MS/MS 703.7 [44] (ion trace not displayed in Figure 7).

The LC-chromatography for negative (Figure 6) and positive (Figure 7) APCI-IT-MS/MS resulted in the detection of different components. This is depending on the ionization capacities of the respective molecules. Hydrocarbon based compounds, such as phytosterols, triterpenoids and beewax compounds resulted in better ion yields in positive ionization mode (Figure 7). Phenolic structures such as artepillin C (Figure 6) were better suitable for negative ionization, as seen in the LC-APCI-IT-MS/MS runs with identical parameters for LC-chromatography.

The LC-APCI-IT-MS/MS chromatography (positive mode) detected for the selected ion trace [M+H]^+^ *m*/*z* 409 a large variety of chromatographically not sufficiently resolved components (Figure 7) which could correspond to a variety of already known phytosterols and triterpenoids such as lupeol, olean-18-ene-3-ol, β-amyrin, lanosterol, 9,19-cyclolanost-7-ene-3-ol and 4,14-dimethyl-ergosta-5,24-diene-3-ol [43]. APCI ionization might have induced rapid cleavage of hydroxyl groups leading to [M-H_2_O+H]^+^ signals.

### 4.4. Morphological Characterization of the GPSE-γCD Complex by Scanning Electron Microscopy

Scanning electron microscopy was used (S-4500, HITACHI, Tokyo, Japan) to characterize the morphology of the GPSE-γCD complex. For the analysis, GPSE-γCD complexes were sprinkled onto conductive glue on a palladium scanning electron microscopy stub and sputter coated with Au/Pd (60/40) for 1 min. Then, the GPSE-γCD complex was analysed at 15 kV for morphology analysis. Three different fields within each sample were randomly chosen, and three images of each field were measured at the magnifications 1000, 3000 and 5000. As depicted in Figure 1C–E, the GPSE-γCD complex exhibited a relatively variable and non-uniform particle shape and size.

### 4.5. Blood Glucose and Body Composition

At the beginning of the trial and at week 7, blood was collected from the tail tip from five mice from each experimental group prior to a 5–6 h fast, and blood glucose levels were determined (Glucometer, Abbott Freestyle Lite, Wiesbaden, Germany).

On week 8, the body composition was measured using the time-domain nuclear magnetic resonance technique (MiniSpec, Bruker, BioSpin MRI GmbH, Ettlingen, Germany) and the following parameters: X-ray energy settings: 45 kVp and 177 µA; voxel size: 76 µm; integration time: 300 ms; and projection setting: 250 projections per 180°. Fat mass, lean mass and free water weight were obtained within two minutes in the live animals.

### 4.6. Hepatic Gene Expression Using Quantitative Reverse Transcriptase PCR

RNA was isolated and purified using a microRNA NucleoSpin^®^ Kit (Macherey & Nagel, Düren, Germany). The concentration and purity of the isolated RNA was measured and controlled using a NanoDrop spectrophotometer (Thermo Scientific, Peqlab Biotechnologie GmbH). The primers for qRT-PCR were designed using Primer3 Input software (v. 0.4.0, Whitehead Institute for Biomedical Research, Cambridge, MA, USA, Table S2) and purchased from Eurofins MWG (Ebersberg, Germany). One-step qRT-PCR was performed using a SensiMix™ SYBR No-ROX One-Step Kit (Bioline, Luckenwalde, Germany), including SYBR Green detection on a RotorGene 6000 cycler (Corbett Life Science, Sydney, Australia). The relative mRNA levels were calculated with an external standard curve using the average expression levels of housekeeping genes (Rn18S or β-actin).

### 4.7. MicroRNA Determination

TaqMan microRNA Assay kits (mmu-miR-146a, mmu-miR-34a) were obtained from Applied Biosystems ABI, Foster City, CA, USA. Quantification of microRNA was performed as a two-step RT-PCR. Reverse transcription reaction was performed with specific microRNA primers targeting miR-146a and miR-34a. Real-time PCR amplification was carried out using a RotorGene 6000 cycler (Corbett Life Science, Sydney, Australia) under standard conditions. Relative microRNA concentrations were determined from the ratios between the amount of the target microRNA and the endogenous mouse control snoRNA-202.

### 4.8. Determination of Hepatic Protein Expression Levels by Western Blotting

Protein expression was determined in the cytosolic lysates (TGF-β1) or in the nuclear fraction (Nrf2, p65) prepared from fresh liver tissues. A total of 40 μg protein from each sample was mixed with loading buffer, denatured at 95 °C for 5 min and separated by a SDS-PAGE Precast Gel (BioRad, Munich, Germany). The fluorescence of the proteins was activated by UV-exposure for 5 min before transferring the proteins onto a polyvinylidene difluoride (PVDF) membrane (BioRad). Proteins were identified using respective primary (Nrf2: 1:200, sc-722, Santa Cruz Biotechnology, Heidelberg, Germany; NF-κB p65: 1:200, sc-373, Santa Cruz Biotechnology; TGF-β1: 1:1000, ab92486, abcam, Cambridge, UK) and secondary antibody (1:4000, GAR-HRP, BioRad) and visualized with enhanced chemiluminescence (ECL) reagents (Fisher Scientific, Schwerte, Germany) in a ChemiDoc XRS system (BioRad). The band intensities were calculated using the Image Lab 4.1 Software (BioRad). The relative protein expression was calculated from the total protein loaded per lane. Prior to data analysis, the critical protein load was determined to avoid saturated fluorescence signals by establishing a dose-dependent relationship between the protein loaded and the fluorescence signal [45].

### 4.9. Statistical Analyses

Results are given as the means ± standard deviations (SD). Significant differences were determined by testing for linear trends using one-way analysis of variance (ANOVA), followed by the Bonferroni correction or, in the case of inhomogeneity of variance, by Dunnett’s multiple comparison post hoc test. The results of body weight and food intake measurements were analysed by two-way ANOVA (factor: time and diet) followed by the Bonferroni multiple comparison post hoc test. *p*-Values less than 0.05 were considered significant. Statistical analyses were performed with GraphPad Prism (Version 7.02, GraphPad Software, La Jolla, CA, USA).

## 5. Conclusions

Natural compounds such as Brazilian green propolis are being increasingly recognized as potential anti-inflammatory compounds. It can be concluded from this study that GPSE-γCD exhibits anti-inflammatory properties and has the potential to serve as a natural bioactive compound to counteract chronic inflammatory processes. Present findings on the anti-inflammatory properties of GPSE-γCD in laboratory mice should be validated in human intervention studies in the future.

## Figures and Tables

**Table 1:** Composition of the experimental diets.

Ingredients	Western-Type Diet (WTD)
Macronutrients	
Crude protein	17.5%
Crude fat	21.2%
Crude fibre	5.0%
Crude ash	4.5%
Nitrogen-free extracts	48.8%
Starch	14.6%
Sugar	33.2%
Cholesterol	2.1 mg/kg
Supplements	
γ-Cyclodextrin (WTD+γCD)	2.2 g/kg
Green propolis supercritical extract-γCD (WTD+GPSE-γCD) ^1^	2.3 g/kg

^1^ providing 200 mg artepillin C per kg diet.

## Supplementary Materials

Supplementary materials can be found at www.mdpi.com/1422-0067/18/6/1140/s1.

## Abbreviations

CAPE	Caffeic ester phenethyl ester
γCD	γ-Cyclodextrin
GPSE	Brazilian green propolis supercritical extract
Sap	Serum amyloid P
SD	Standard deviation
LPS	Lipopolysaccharide
Tlr	Toll-like receptor
TGF-β1	Transforming growth factor β1
TNF-α	Tumour necrosis factor α
WTD	Western-type diet
