# Supplementary material for: Anti-Inflammatory Properties of Brazilian Green Propolis Encapsulated in a γ-Cyclodextrin Complex in Mice Fed a Western-Type Diet
Source: Int J Mol Sci. 2017 May 26;18(6):1140. doi: 10.3390/ijms18061141 (PMC5485965; doi:10.3390/ijms18061141)
Supplement: Supplementary file 1 [file ijms-18-01141-s001.pdf]

Supplementary File:

Table S1. Body weight and food intake over 10 weeks in mice fed a Western-type diet (WTD), or a WTD supplemented with either γ-cyclodextrin (γCD) or green propolis supercritical extract encapsulated in γCD (GPSE-γCD).

week	0	1	2	3	4	5	6	7	8	9	10	ANOVA
Body weight (g)												
WTD	19.2±1.15	19.4±1.48	20.6±1.93	20.8±2.19	21.6±1.99	22.5±2.21	22.4±2.28	22.9±2.79	23.7±2.82	24.1±2.75	23.6±3.38	
γCD	19.2±0.99	19.6±1.22	20.9±1.35	21.7±1.77	22.0±1.27	22.7±1.48	23.5±1.64	23.5±2.00	23.7±1.69	24.6±2.18	24.4±2.31	**time: p<0.001**
GPSE-γCD	19.3±0.93	19.5±1.20	20.6±1.02	21.3±1.21	21.6±1.20	22.6±1.21	23.0±2.03	23.0±1.96	24.1±1.58	24.5±2.07	24.7±2.04	**diet: ns**
Food intake (g/mouse/day)												
WTD		2.45±0.44	2.40±0.14	2.29±0.19	2.42±0.18	2.42±0.15	2.30±0.15	2.46±0.16	2.49±0.25	2.43±0.16	2.28±0.27	
γCD		2.46±0.40	2.25±0.13	2.29±0.19	2.26±0.29	2.47±0.67	2.28±0.23	2.33±0.16	2.23±0.25*	2.48±0.24	2.10±0.26	**time: p<0.001**
GPSE-γCD		2.30±0.75	2.29±0.22	2.42±0.14	2.44±0.19	2.51±0.29	2.34±0.26	2.37±0.24	2.43±0.26	2.55±0.27	2.29±0.23	**diet: p<0.01**

The data are presented as the means±SDs in n=10 mice/diet. Significant differences were calculated using two-way ANOVA (factor: time and diet), followed by the Bonferroni multiple comparison post hoc test. ns=not significant * p<0.05 compared to WTD within one week.

Table S2. Nucleotide sequences and annealing temperatures of primers used in qRT-PCR analyses.

Gene	Gene-ID	Description	Primer, Forward (5′-3′)	Primer, Reverse (5′-3′)
Tnf-α	21926	tumour necrosis factor-α	TCGTAGCAAACCACCAAGTG	AGATAGCAAATCGGCTGACG
Sap	20219	serum amyloid P	AAGCTGCTGCTTTGGATGTT	CATTGTCTCTGCCCTTGACA
Il1b	16176	interleukin-1 beta	CAGGCAGGCAGTATCACTCA	AGCTCATATGGGTCCGACAG
iNOS	18126	inducible nitric oxide synthase	GGCAGCCTGTGAGACCTTTG	GCATTGGAAGTGAAGCGTTTC
Tlr2	24088	toll-like receptor 2	GCATCCGAATTGCATCACCG	CCTCTGAGATTTGACGCTTTGTC
Tlr4	21898	toll-like receptor 4	TCAGAACTTCAGTGGCTGGA	GAGGCCAATTTTGTCTCCAC
Gclc	14629	glutamate cysteine ligase, catalytic subunit	GTGGAGGCCAATATGAGGAA	GGGTGCTTGTTTATGGCTTC
Gclm	14630	glutamate cysteine ligase, modifier subunit	TCCCATGCAGTGGAGAAGAT	AGCTGTGCAACTCCAAGGAC
Pon1	18979	paraoxonase 1	CAGCCTGTCCATCTGTCTCA	CACCCGTCTCGATTCCTTTA
Cat	12359	catalase	GGAGCAGGTGCTTTTGGATA	CTGACTCTCCAGCGACTGTG
Gpx4	625249	glutathione peroxidase 4	ATGAAAGTCCAGCCCAAGG	CGGCAGGTCCTTCTCTATCA
Nrf2	18024	nuclear factor 2	GCAACTCCAGAAGGAACAGG	GCAATGTCTCTGCCAAAAGC
Ftl1	14325	ferritin light	CTTCCAGGATGTGCAGAAG	ATCCAAGAGGGCCTGATT
Hepc	84506	hepcidin	GCACCACCTATCTCCATCA	GGGGAAGTTGGTGTCTCTC
Mt1	17748	metallothionein 1	ACCTCCTGCAAGAAGAGCTG	GCTGGGTTGGTCCGATACTA
